# Lateral gene transfer in eukaryotes: tip of the iceberg or of the ice cube?

**DOI:** 10.1186/s12915-016-0330-x

**Published:** 2016-11-18

**Authors:** Etienne G. J. Danchin

**Affiliations:** Institut Sophia Agrobiotech, INRA, University of Nice Sophia Antipolis, CNRS, 06903 Sophia Antipolis, France

## Abstract

Lateral gene transfer (LGT) is the transmission of genes, sometimes across species barriers, outwith the classic vertical inheritance from parent to offspring. LGT is recognized as an important phenomenon that has shaped the genomes and biology of prokaryotes. Whether LGT in eukaryotes is important and widespread remains controversial. A study in *BMC Biology* concludes that LGT in eukaryotes is neither continuous nor prevalent and suggests a rule of thumb for judging when apparent LGT may reflect contamination.

See research article: http://bmcbiol.biomedcentral.com/articles/10.1186/s12915-016-0315-9.

## Importance of lateral gene transfer in prokaryotes and eukaryotes

Lateral (or horizontal) gene transfer (LGT) refers to the transmission of genes between individuals without direct vertical inheritance from parents to their offspring. In contrast to vertical inheritance, LGT can cross species barriers and may even allow transmission of genes across the kingdoms of life. In prokaryotes, LGT is well documented and the supporting mechanisms have been widely described [1].

The high prevalence of LGT in prokaryotes has even challenged the validity of a bifurcating Darwinian tree of life and led to the suggestion that an interconnected rhizome of life would be a more realistic representation of relations between species [2]. The model bacterium *Escherichia coli* is a good illustration of the plasticity of bacterial gene repertoires due to gene acquisition via LGT and differential loss. While a typical *E. coli* genome contains ~5000 protein-coding genes, the pan-genome of *E. coli*, taking into account more than 60 different strains, is estimated to contain more than 15,700 genes [3]. Interestingly, only 6 % of these genes are present in every strain and the variable portion of a typical *E. coli* gene set reaches 80 %. Furthermore, LGT plays crucial roles in acquisition of antibiotic resistance, adaptation to new environments, and pathogenicity of bacteria. Hence, it is commonly accepted that the biology, genome composition, and ecology of prokaryotes have been deeply impacted by LGT.

**Fig. 1:**
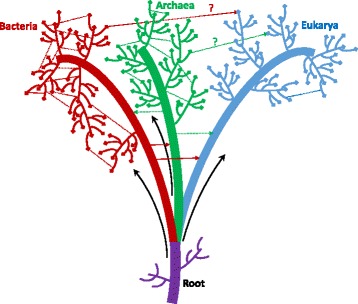
How prevalent is LGT in prokaryotes and eukaryotes? Schematic representation of the cellular organism tree of life. The root of the tree is indicated in *violet* and Bacteria, Archaea, and Eukarya branches in *red*, *green*, and *blue*, respectively. *Plain black arrows* represent vertical gene inheritance from ancestors to their descendants. *Dashed lines* represent LGT events. Within Bacteria and Archaea, the branches are highly interconnected by LGT events. In Eukaryotes there is much less interconnection and recent gene acquisition of prokaryote origin via LGT is controversial. *Violet branches* emerging from the root represent extinct clades

In eukaryotes, there is no clear description of a mechanism for straightforward horizontal gene exchange between species. Furthermore, there has been no report of abundant eukaryote to eukaryote LGT to date (Fig. 1). Although fungi are known to exchange conditionally dispensable whole chromosomes, and that this can affect their pathogenicity, this seems to be restricted to strains of the same or closely related species [4].

Prokaryote to eukaryote LGT intuitively appears to be even less straightforward. Prokaryotic genes are subject to transcriptional and translational controls that are quite different from those of eukaryotic genes. Hence, even if a prokaryotic gene were successfully integrated in a eukaryotic genome, functional assimilation would be difficult. These complications are amplified in metazoan species, in which the germline is usually separated from the rest of the cells. A gene acquired from a prokaryote would have no chance of being fixed at the level of a population or a species if it were not transmitted to the next generation through integration into the germline. Analysis of somatic human samples suggests the separate germline is actually a stringent barrier against LGT [5].

## Claims of extensive LGT in eukaryotes are controversial

Yet, despite all these barriers, there have been repeated claims of extensive LGT from prokaryotes to eukaryote nuclear genomes, including in metazoans (see [6] and references therein). It has been proposed that none of these barriers is insurmountable and that we may even underestimate the contribution of LGT to the biology and genomes of eukaryotes. However, despite the well accepted LGT from mitochondria and plastids to the nuclear genome, whether prokaryotes have significantly contributed to eukaryote genomes via LGT remains a matter of dispute. A recent controversy about the prevalence of prokaryote to eukaryote LGT is the tardigrade case (see [7] and references therein). An initial analysis of a draft tardigrade genome suggested that LGT contributed to up to ~17 % of the gene set. Although far from the contribution of LGT to the genomes of prokaryotes, this represented the highest proportion of genes acquired by lateral transfer in an animal so far. Soon after, another report of an independent genome assembly refuted this result and suggested that only 1–2 % of genes have been laterally acquired in the tardigrade and that the huge difference was probably due to bacterial contamination mistakenly attributed to genes acquired by LGT. A third analysis, in which tardigrades were treated with antibiotics and starved before sequencing, and in which remaining contaminants were removed, concluded that the contribution of LGT to the gene set was probably 4–5 %. To date, the second-highest reported proportion of LGT for a eukaryote was 8–9 % for the bdelloid rotifers, with the same uncertainty as for the tardigrades. Thus, it remains unclear whether LGT of prokaryotic origin has contributed significant proportions of eukaryotic gene sets.

## Have LGT of prokaryotic origin significantly contributed to current eukaryotic genes sets?

The extent of prokaryotic LGT to eukaryotes is the question that Ku and Martin decided to tackle in their recent article in *BMC Biology* [8]. Their idea is that if LGT from prokaryotes to eukaryotes is continuous and prevalent, traces of recent LGT must be detectable in eukaryote genomes. To assess this, they have re-analyzed their 2015 dataset made up of ~2600 phylogenetic trees encompassing 55 eukaryotes from diverse lineages and ~2000 prokaryote species. While they identify many prokaryote to prokaryote LGT candidates with high similarity between donor and receiver genes, indicative of recent transfer, they found a paucity of highly similar prokaryote to eukaryote LGT candidates. Moreover, while in prokaryotes recent LGT candidates are present in multiple species in the receiver clade, this is much more rarely observed in eukaryotes. Furthermore, if the candidate eukaryotic acquisitions from plastid and mitochondrial ancestors are excluded from the analysis, there remain only a few species-specific recent candidate LGT events. Because these few recent candidates are specific to one or a few species and highly similar to their prokaryotic candidate donors, they cannot easily be distinguished from bacterial contamination. On the basis of these observations, the authors conclude that there is a lack of evidence for recent LGT of prokaryotic origin in eukaryotic genomes and that this phenomenon is neither continuous nor prevalent. They further propose that any protein-coding gene in a eukaryotic genome with ≥70 % identity to prokaryotic homologs should be first considered as likely contamination rather than candidate LGT.

Obviously, several confounding factors could also contribute to this paucity of candidate recent LGT in eukaryote genomes. First, in their dataset, the authors include almost 40 more bacterial species (including closely related species or different strains of the same species) than eukaryotic species (none of which are closely related). This can partly contribute to the paucity of recent candidate LGT conserved between multiple eukaryote species within a receiver clade. One could also argue that true candidate prokaryotic LGT donors have not been sampled because most are probably uncultured bacteria distant from anything that has been sequenced. Finally, the removal of everything highly similar to bacterial genes prior to eukaryotic genome annotation (or assembly) could also contribute to this deficiency of putative recent LGT. In many genome projects these highly similar sequences are considered as contaminants and are not visible in the final set of predicted protein-coding genes. However, as stated by the authors, these features probably account for only a minor part of the huge difference between prokaryote–prokaryote and prokaryote–eukaryote distribution of similarity between candidate donor and receiver genes. It is almost certain that the contribution of LGT of prokaryote origin to the making of a eukaryotic nuclear genome is several orders of magnitude less important than for prokaryotes.

What we can conclude from this recent paper and the tardigrade controversy is that any claim of prokaryote–eukaryote LGT (and particularly those with high identity to prokaryote candidate donors) must be taken with caution and, ideally, additional supporting evidence should be gathered. In addition to phylogenetic analysis, features such as presence of bona fide eukaryotic genes on the same contigs as the candidate LGT sequences, the presence of spliceosomal introns, conservation of the LGT candidate in sister species, and transcriptional support all provide additional evidence for LGT rather than contamination.

## The contribution of LGT to eukaryote genomes can still be biologically significant

Even if the concept of a pan-genome shaped by LGT and differential gene loss certainly does not apply as a general rule in eukaryotes, this does not necessarily mean that prokaryote to eukaryote LGT is insignificant for their biology. One of the clearest examples has come from research that we and other colleagues have developed on plant-parasitic nematodes and insect herbivores. These animal species have acquired a whole set of enzymes from bacteria to help them degrade the plant cell wall, other plant poly- and oligo-saccharides, or to overcome plant defense mechanisms [9, 10]. Although these gene acquisitions do not contribute a high proportion of their gene sets, they play very important roles in the biology of these species. In the nematodes, these genes are transcribed in secretory organs, the enzymes are found in the nematode secretions, and inactivation of the genes via RNA interference reduces the efficiency of parasitism.

In some cases the enzymes are present both in the nematodes and the insects and the phylogenies show that they form separate groups, each of which is related to a different group of bacteria or other non-metazoan homologs [11]. A common ancestral acquisition followed by multiple gene losses appears very unlikely to explain these topologies and multiple independent acquisitions is the most straightforward hypothesis so far.

Although LGT events are not as prevalent and have certainly not contributed as significantly to the genome composition of eukaryotes as they have to those of prokaryotes, they still represent evolutionarily and biologically significant events, including in animals. As further genomes and a more substantial portion of the biodiversity is sampled, new intriguing LGT cases, including from eukaryote to eukaryote or even eukaryote to prokaryote (or virus), will probably emerge and continue to feed discussion and debate among evolutionary biologists.
